# Refining a multimedia patient-reported outcomes measure for patients with low literacy

**DOI:** 10.1186/s41687-026-01081-6

**Published:** 2026-05-14

**Authors:** Chao Long Azad, Aygul Iskandarova, Caroline Wu, Laura Beres, Albert W. Wu, Allan Fong, Aviram Giladi

**Affiliations:** 1 The Curtis National Hand Center, MedStar Union Memorial Hospital, Baltimore, USA; 2 Department of Medicine, Roger Williams Medical Center, Providence, USA; 3 Department of Orthopaedic Surgery, Duke University, Durham, USA; 4 Department of International Health, Johns Hopkins Bloomberg School of Public Health, Baltimore, USA; 5 Department of Health Policy and Management, Johns Hopkins Bloomberg School of Public Health, Baltimore, USA; 6 MedStar Health Research Institute, Columbia, USA

**Keywords:** Healthy literacy, Human-centered design, Instrument development, Measurement equity, Patient-reported outcomes measures, Qualitative evaluation, Usability testing

## Abstract

**Background:**

Patients with low literacy have difficulty completing patient-reported outcomes measures (PROMs). The Multimedia Adaptation Protocol (MAP) guides the process of adapting traditional PROMs to multimedia versions that patients with low literacy can self-administer. We previously executed the first MAP stage, forward adaptation, to adapt the Patient-Reported Outcomes Measurement Information System Upper Extremity (PROMIS-UE) to a multimedia version (multimedia PROMIS-UE). The goal of this study was to refine the multimedia PROMIS-UE via the next MAP stages, back adaptation and qualitative evaluation, to result in a conceptually-equivalent, user-tested multimedia PROMIS-UE ready for psychometric validation.

**Methodology:**

For back adaptation, mixed-literacy and English-speaking participants masked to the PROMIS-UE “translated” multimedia components back to text. We compared text back-adaptations to the original PROMIS-UE, identified areas of non-equivalence, and updated the multimedia PROMIS-UE accordingly. For qualitative evaluation, we used an iterative design process to conduct cycles of testing to assess usability and face validity. We performed analyses including memo writing and framework-guided thematic analysis. Findings were synthesized into usability, functionality, or content-related themes that guided ideation workshops focused on identifying solutions.

**Results:**

We recruited 11 participants for back adaptation. We identified areas of non-equivalence between the PROMIS-UE and multimedia PROMIS-UE for 85% of the questions, which were addressed in an updated multimedia PROMIS-UE. We conducted two cycles of qualitative evaluation. We recruited 20 patients and identified 16 themes/challenges: animations unused, audio unused, instructions unclear, instructions unused, difficulty starting questionnaire, response audio button misinterpreted, difficulty with incomplete forms, difficulty submitting answers, purpose of avatars unclear, purpose of smiley scale unclear, difficulty differentiating smileys, inaccurate animation, unclear how to select answer, audio button unused or misinterpreted, confusion with scrolling, and incomplete popup window missed. We updated the multimedia PROMIS-UE after each cycle to address each theme.

**Conclusions:**

We developed a conceptually-equivalent, user-tested multimedia PROMIS-UE ready to undergo psychometric testing and validation. This work represents an essential intermediary and preparatory phase of PROM development, is analogous to cross-cultural translation phases intentionally positioned prior to psychometric validation, and advances *how* PROMs can be adapted to a multimedia format. Ultimately, a validated multimedia PROMIS-UE will expand clinicians’ and investigators’ abilities to capture patient-reported outcomes in low literacy populations.

## Background

In the United States (US), low literacy can be defined as reading proficiency at or below the sixth-grade level, as this limits the ability to understand written health information and complete written questionnaires independently [1–3]. It is estimated that between one-fifth and almost half of all adults have low levels of English literacy [4, 5]. Low literacy is independently associated with poor health status [6], and rates of low literacy are disproportionately higher among cohorts with challenges in social determinants of health (SDOH), often including racial and ethnic minority populations [4, 5, 7]. Although measuring patient-reported outcomes (PROs) has become a critical component of understanding and improving care and health status, administration of patient-reported outcomes measures (PROMs) in low literacy patient populations remains a major challenge [8]. This aligns with recent studies that have shown that PROM completion rates are influenced by SDOH [9, 10].

Almost all existing PROMs rely on text to convey meaning [11]. This precludes an accurate measurement of outcomes for low literacy populations, prevents providers from comprehensively evaluating these vulnerable populations at the point of care, and limits their inclusion in research. Although interviewer administration of PROMs is an option when self-administration is not feasible, it can be labor intensive and expensive, making it impractical and cost prohibitive as PROs become widely used [12]. Interviewer administration also introduces the potential for interviewer bias [12] and patient embarrassment [3], important considerations for patients with low literacy who may hide the inability to read due to shame [13, 14]. Growing reliance on PROs to understand outcomes facilitates the provision of patient-centered care, and measure quality can result in these tools contributing to the perpetuation and exacerbation of health disparities unless PROMs are designed for inclusive and equitable implementation [15].

**Fig. 1:**
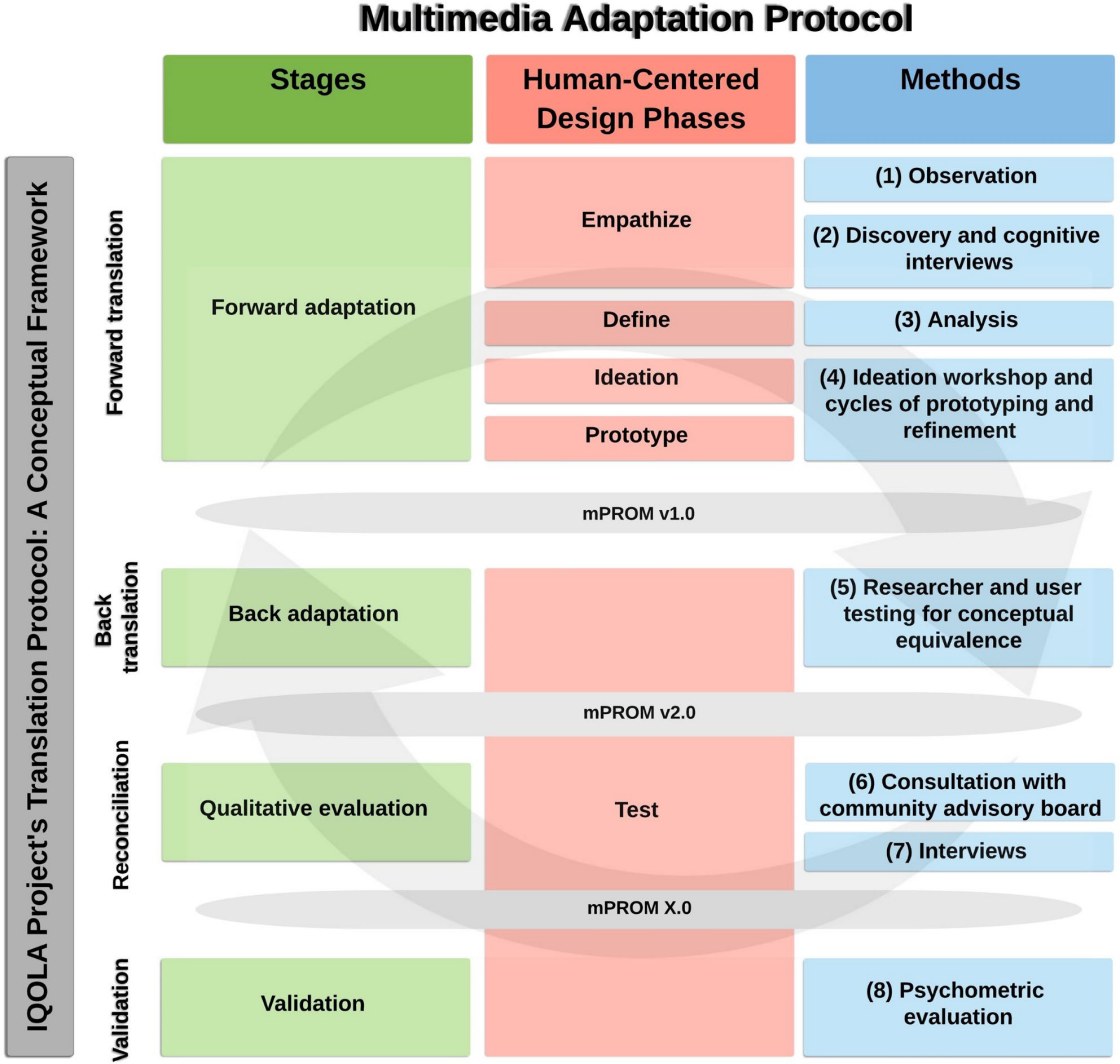
**Multimedia adaptation protocol (MAP). **The IQOLA project translation protocol was used as a guiding conceptual framework to develop the MAP, which included four stages (green): (1) forward adaptation, (2) back adaptation, (3) qualitative evaluation, and (4) validation. This manuscript reports the findings from the back adaptation and qualitative evaluation stages. The corresponding human-centered design phases and methods are shown in red and blue, respectively. (IQOLA = International Quality of Life Assessment; MAP = Multimedia Adaptation Protocol; HCD = human-centered design; mPROM = multimedia patient-reported outcomes measure; v1.0 = version 1.0; v2.0 = version 2.0; X.0 = final version). Reproduced from Long C, Beres LK, Wu AW, Giladi AM (2021) Developing a protocol for adapting multimedia patient-reported outcomes measures for low literacy patients. PLoS One 16(6):e0252684 10.1371/journal.pone.0252684, under the terms of the Creative Commons CC BY 4.0 license. No changes were made to the original figure

We developed the Multimedia Adaptation Protocol (MAP) to guide translation of text-only PROMs to multimedia PROMs (mPROMs) with audio and video components that can be self-administered by patients of all literacy levels (not to be confused with mHealth (mobile health) technologies) [16]. The MAP takes a human-centered design (HCD) approach and has four stages: (1) forward adaptation, (2) back adaptation, (3) qualitative evaluation, and (4) psychometric validation (Fig. 1) [16]. For the first development of a mPROM, we focused on the hand and upper extremity population because we expected variation in physical function to be easier to convey in multimedia elements than variation in mental or social health. We used (1) forward adaptation to adapt the Patient-Reported Outcomes Measurement Information System Short Form v2.0 Upper Extremity 7a (PROMIS-UE) to a multimedia prototype (multimedia PROMIS-UE) [17]. We selected PROMIS-UE because PROMIS instruments are among those that the National Institutes of Health encourages investigators to use [18] and because the PROMIS-UE item bank affords us flexibility to create a customized short form if needed [19–21]. Additionally, although PROMIS development standards specify that items should be written at a sixth grade reading level or lower [22], we calculated that the PROMIS-UE Short Form v2.0 Upper Extremity 7a including the instructions has a SMOG (Simple Measure of Gobbledygook) reading level of the seventh grade. The goal of this study was to refine the multimedia PROMIS-UE via (2) back adaptation and (3) qualitative evaluation to produce a conceptually-equivalent, user-tested multimedia PROMIS-UE ready to undergo (4) psychometric validation.

## Methods

### Study setting

This study was conducted with hand and upper extremity patients recruited from the Curtis National Hand Center (CNHC). CNHC has broad experience and a strong infrastructure to support PRO data collection and management with > 90% of patients completing data entry and questionnaires [23]. Despite overall high completion rates, we found that patients with a high school education or less, who are more likely to have lower levels of literacy, are less likely to complete PROs before their clinic visit [23]. Additionally, CNHC is located in Baltimore City, Maryland, a city with rates of illiteracy [24] and poverty [25] above the national average. CNHC’s PRO-focused efforts and experience in a city with substantial health services needs make it an ideal site for developing and refining a mPROM aimed at improving PRO collection in patients with low literacy.

### Multimedia PROMIS-UE prototype

**Fig. 2:**
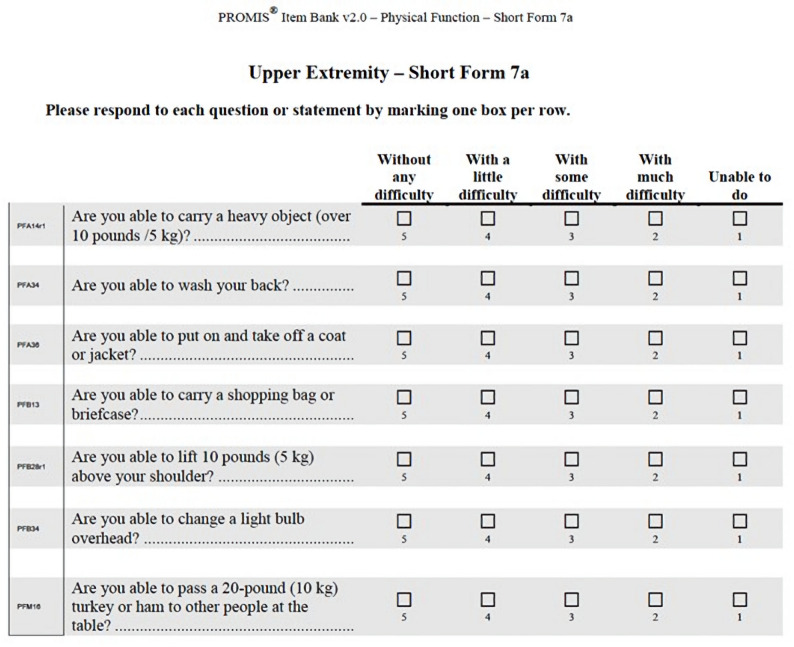
**PROMIS-UE v2.0 Short Form 7a. **Patient-reported outcomes measurement information system upper extremity (PROMIS-UE) Short Form 7a is the patient-reported outcomes measure that we adapted to a multimedia version

**Fig. 3:**
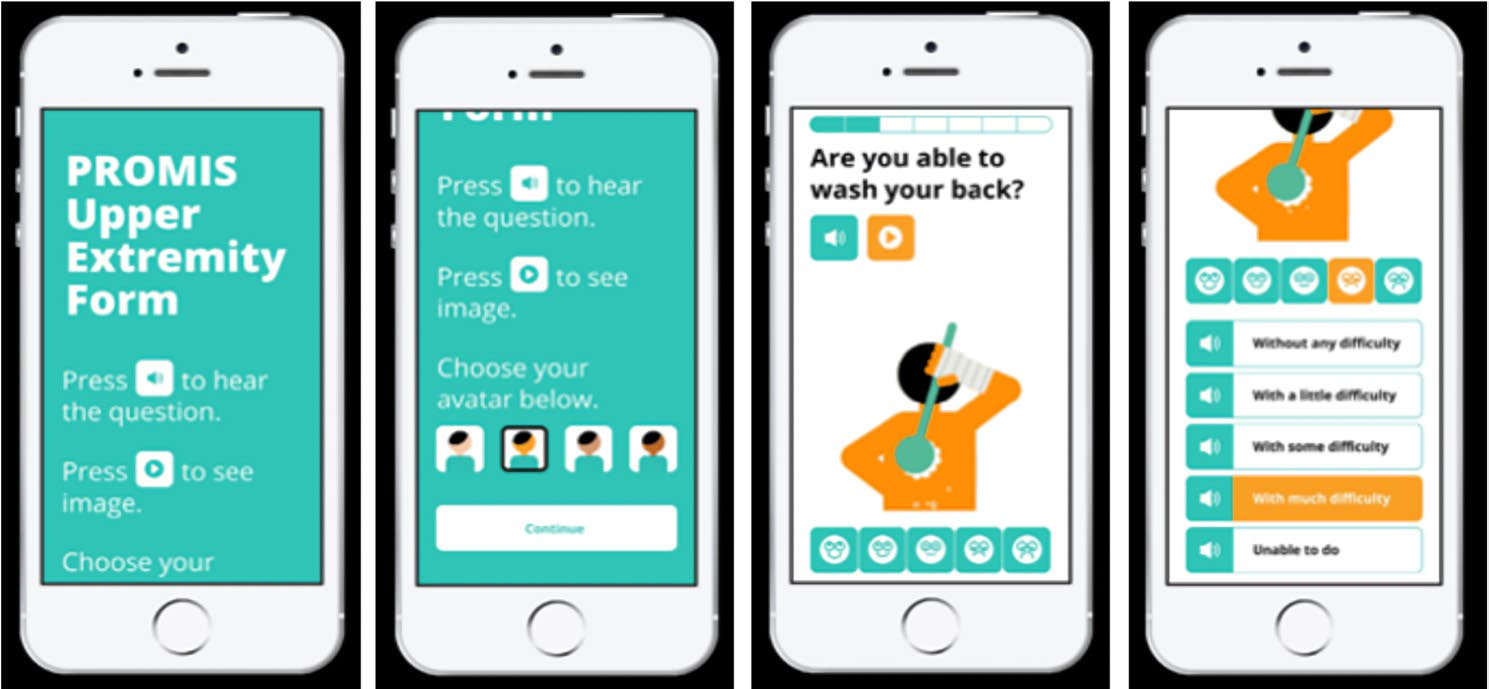
**Screen captures of the multimedia PROMIS-UE Prototype. **This prototype has features including audio, animations (graphics interchange format [GIFs]), an illustrated response scale, icons, a progress indicator, and avatars. This prototype was the starting point for the research reported in this manuscript and can be viewed online [17]. Reproduced from Azad CL, Beres LK, Wu AW, Fong A, Giladi AM (2024) Developing a multimedia patient-reported outcomes measure for low literacy patients with a human-centered design approach. PLoS One 19(6):e0304351. 10.1371/journal.pone.0304351, under the terms of the Creative Commons CC BY 4.0 license. No changes were made to the original figure

We performed forward adaptation by adapting PROMIS-UE Short Form 7a (Fig. 2) to a high-fidelity multimedia PROMIS-UE prototype [17], which can be viewed online [26] or seen in representative screenshots (Fig. 3). The prototype’s features included audio, animations (graphics interchange format [GIFs]), an illustrated response scale, icons, a progress indicator, and avatars. This prototype was the starting point for the research reported in this manuscript.

### Back adaptation

The goal of back adaptation is to ensure conceptual equivalence, defined as the adapted instrument (i.e., the multimedia PROMIS-UE) conveying the same content as the source instrument (i.e., the PROMIS-UE) [16]. This stage is needed to ensure that we have not altered the content of the PROMIS-UE during forward adaptation. We recruited English-speaking adult participants who had never been exposed to the PROMIS-UE. Participants included members of the CNHC team unfamiliar with this work as well as mixed-literacy patients being evaluated or treated for upper extremity issues. We aimed to recruit approximately 10 participants total per our HCD approach [27].

For the entire cohort, we conducted structured interviews during which participants “translated” the multimedia components back to text. We did this for two versions of the multimedia PROMIS-UE: first, a version of the multimedia PROMIS-UE with only the visual components (i.e., the animations/GIFs and the illustrated response scale, of which each animation and each graphic of the illustrated response scale were “translated” separately), and then a version with both visuals and audio. This approach ensured that participants remained masked to the text and the audio “reading” of the text when performing back-translation of the visuals. For the patient participants, we also conducted semi-structured interviews to identify and understand areas of confusion stemming from the questions, response options, visuals, or audio components. These semi-structured interviews ended with an assessment of patients’ literacy levels with the Rapid Estimate of Adult Literacy in Medicine Short Form (REALM-SF); a score of 6 or less, corresponding to a sixth-grade reading level or less, indicated low literacy [1, 2].

Back adaptation of the translated text was used to compare to the original PROMIS-UE, with the goal of identifying areas of nonequivalence. We conducted framework-guided rapid thematic analysis [28, 29] of the data from the semi-structured interviews. The research team then convened to discuss findings with the goal of reaching a consensus on how the instrument should be changed to address areas of nonequivalence and/or other identified issues. Our illustrator updated multimedia components accordingly, and our technical team incorporated these updated assets into the next version of the multimedia PROMIS-UE. These updated multimedia components were retested in the following qualitative evaluation stage.

### Qualitative evaluation

The goal of this stage was to understand the “true” user experience and user comprehension, so that we could identify design and content-related challenges. We recruited English-speaking mixed-literacy adult patients from CNHC. We used an iterative design process, which has been shown in HCD literature to be rigorous yet efficient [30, 31]. We ended testing once we reached saturation of usability, functionality, and content-related themes. Usability refers to ease of navigation and interaction with the interface, whereas functionality refers to whether interface elements performed as intended.

Patients underwent testing in four stages. (1) *Observation and ‘Think Aloud’*: We observed patients completing the multimedia PROMIS-UE with no guidance to simulate a real user environment. As they were doing this, they were asked to “think aloud,” a cognitive interview technique that allowed us to gather patients’ thoughts and understand their thought processes while engaging with the multimedia PROMIS-UE [32]. (2) *Design*: Using semi-structured interview guides, we elicited design-related issues that compromised usability and clarity, including questions/prompts that targeted issues observed from Part 1 and prior cycles of testing. In the final cycle, we performed A/B testing, a widely-used effective and efficient method for comparing product variants [33], to test variations of a solution for a given problem. (3) *Forced Multimedia*: Because all of the multimedia components in our prototype were optional (i.e., they were only deployed if the user clicked on the button), some participants did not see any of the multimedia components built into the prototype. The interviewer showed these participants the multimedia components followed by a set of questions aimed to identify reasons why the multimedia components were not used, elicit feedback on the multimedia features, and understand how the multimedia components affected user experience and understanding. (4) *Content*: We asked cognitive interview questions that aimed to identify content-related issues that compromised understanding. To conclude user testing, we assessed all patients’ literacy levels with the REALM-SF. All sessions were audio-recorded with the patients’ permission and transcribed to facilitate analysis.

We conducted initial and intermediate analyses that assessed usability and face validity. Initial analyses were deductive in nature and performed by the interviewer (AI). This included analytic memo writing and framework-guided thematic analysis. One framework was the 14 Nielsen-Schneiderman Heuristics [34], which were used to conduct a usability heuristic evaluation (a technique that identifies usability problems in a user interface design [35]). The findings from the initial analyses then underwent intermediate analyses that included reviewing all data (audio recordings, transcripts) and initial analyses (memos, deductive frameworks), performing a check for inductive themes, and synthesizing all findings into usability, functionality, or content-related themes/challenges that needed to be addressed. Analysis culminated in a workshop with our multidisciplinary team, during which we brainstormed potential solutions for each theme/challenge. The solutions that we decided on by consensus were then implemented by our technical team.

## Results

### Back adaptation

We recruited 11 participants for back adaptation. Nearly half of the participants were patients (*n* = 5) and female (*n* = 5). Importantly, for our patient participants (*n* = 5), we successfully recruited a mixed-literacy sample with literacy levels ranging from 1 to 7 and 80% (*n* = 4) of patients with low literacy.

We identified areas of non-equivalence between the PROMIS-UE and multimedia PROMIS-UE for 85% (*n* = 6) of the animations/GIFs and 80% (*n* = 4) of the graphics of the illustrated response scale (summarized in Table 1). All areas of non-equivalence were identified in the animations; none were identified in the audio. These areas of non-equivalence were all addressed in an updated multimedia PROMIS-UE.

**Table 1:** Areas of non-equivalence in visuals (including both the animations/GIFs and the illustrated response scale) and corresponding changes

Visual	Areas of Non-equivalence	Change to Animation
Animation/GIF for “Are you able to carry a heavy object (over 10 pounds/5 kg)?”	(1) Interpreted as 10 pounds rather than as *over* 10 pounds; (2) assumed question included a component of being able to carry something and walk at the same time	(1) “10 lb” changed to “10 lb+”; (2) de-emphasized the walking by making the movement in our animation less brisk and moving the setting from outdoors to indoors; kept some amount of walking due to concerns that it would otherwise be interpreted as “hold” which is conceptually different from “carry”
Animation/GIF for “Are you able to wash your back?”	None	No changes
Animation/GIF for “Are you able to put on and take off a coat or jacket?”	(1) Article of clothing interpreted as a shirt rather than a “coat or jacket”; (2) thought they were asked about ability to put on an article of clothing rather than to put on *and* take off	(1) Added buttons and a collar to better illustrate a “coat or jacket”; (2) added an explicit motion of the jacket being taken off
Animation/GIF for “Are you able to carry a shopping bag or briefcase?”	Assumed the question included a component of being able to carry and walk at the same time	De-emphasized the walking by making the movement less brisk and moving the setting from outdoors to indoors
Animation/GIF for “Are you able to lift 10 pounds (5 kg) above your shoulder?”	Interpreted as lifting above or over your head rather than above your shoulder	Made the object’s movement end above the shoulder rather than above the head; switched to a profile view so that this motion could be better visualized
Animation/GIF for “Are you able to change a lightbulb overhead?”	Interpreted as “reaching” or “putting in” a lightbulb rather than changing a lightbulb	Added a twisting motion so that it was clearer that the lightbulb was being changed; had a light appear after the twisting motion
Animation/GIF for “Are you able to pass a 20-pound (10 kg) turkey or ham to other people at the table?”	Interpreted as “carrying” or “holding” or “sliding” a turkey instead of passing it	Changed from standing to sitting position (better mimicking sitting down at a dinner table, e.g., when you are asked to pass something); added a picking up motion as this is an assumed part of passing something
The five graphics that make up the illustrated response scale	The first and second, and the fourth and fifth graphics were too similar; “they’re both smiling” in reference to the first and second graphics	Graphics of the illustrated response scale were updated so that they were more different and easily distinguishable from one another

Legend: For each question in PROMIS-UE (Column 1), we identified areas of non-equivalence between the PROMIS-UE and multimedia PROMIS-UE versions (Column 2). We then include the changes we made to address those areas of non-equivalence (Column 3)

### Qualitative evaluation

We conducted two cycles of interviews, analysis, and refinement. In the first cycle, we recruited 15 patients who were distinct from those enrolled in the prior back adaptation phase. The mean age was 57 years old, nearly half were female (*n* = 7), and REALM-SF literacy scores ranged from 1 to 8 with 40% (*n* = 6) patients scoring as low literacy. We identified 12 themes/challenges: animations unused, audio unused, instructions unclear, instructions unused, difficulty starting questionnaire, response audio button misinterpreted, difficulty with incomplete forms, difficulty submitting answers, purpose of avatars unclear, purpose of smiley scale unclear, difficulty differentiating smileys, and inaccurate animation for question 5 (“Are you able to lift 10 pounds (5 kg) above your shoulder?”). All themes/challenges were functionality- or usability-related except for the last one regarding the inaccurate animation, which was content-related. We identified and implemented solutions addressing each theme resulting in the next iteration of the multimedia PROMIS-UE. A description of these themes/challenges, in addition to the corresponding changes that were made to the multimedia PROMIS-UE, are summarized in Table 2.

**Table 2:** Themes/challenges from cycle 1 of qualitative evaluation and corresponding changes or options to test in the next cycle

Theme/Challenge	Description	Changes to Multimedia PROMIS-UE and Next Steps
Animations unused	Animations not used/deployed in both low and high literacy cohorts; after animations were demonstrated, most patients liked the feature and wished that they had known about it	Animations play automatically rather than requiring a button to be deployed
Audio unused	Audio not used/deployed in both low and high literacy cohorts; when audio was played, it was unintentional	Change icon for deploying audio to something more intuitive; test four different options to determine which best accomplishes this
Instructions unclear	Instructions did not effectively communicate the availability of multimedia features	Removed the confusing instructions since the above two changes obviated the need for instructions
Instructions unused	Instructions skipped over entirely; this was a large contributor to why animations and audio were unused	Removed the unused instructions since the first two changes obviated the need for instructions
Difficulty starting questionnaire	Had difficulty starting the questionnaire as some patients pressed on things randomly on the instruction page; related to the confusion surrounding the instructions	Removed the instructions page entirely so that the questionnaire doesn’t need to be “started”
Response audio button misinterpreted	Patients thought that the audio button was the way to select their answer rather than a mechanism for deploying audio	Moved the audio button to the right of the text
Difficulty with incomplete forms	Patients were taken back to unanswered questions after clicking “Submit,” but it wasn’t clear to all patients why they were being taken back to the questions and unable to submit	Added an “Incomplete” pop-up after clicking “Submit” if there are incomplete questions; added bright orange highlighting to the text of the incomplete question; highlighting disappears after an answer is selected
Difficulty submitting answers	Had difficulty submitting their answers	Made “Submit” button more noticeable by capitalizing entire word; test “SUBMIT” vs. “FINISH”; test which icon best visually communicates purpose of the button
Purpose of avatars unclear	Did not understand that the chosen avatar would be reflected in the animations; didn’t understand why they were asked to pick one; even after the purpose was explained, felt it was unnecessary	Removed avatars; updated animations to include people with medium skin tone and to be gender neutral; probe in next cycle if any concerns regarding the person in animations
Purpose of smiley scale unclear	Did not understand that each smiley corresponded to a response option; some thought that they were a pain scale or a happiness or emotions scale	Integrated smiley scale with text response (i.e., in the same box) so that the relationship between the smiley and the text response is clearer
Difficulty differentiating smileys	Felt that some of the smileys were too similar and difficult to differentiate	Updated the smiley scale to show more contrast between each smiley
Inaccurate animation	Question 5 states “above your shoulder” but the object being lifted in the animation doesn’t go above the shoulder, only to the level of the shoulder	Updated animation so that the object is lifted above the shoulder

Legend: This table includes the 12 themes/challenges (Column 1) that we identified in our first cycle of qualitative evaluation, as well as a more detailed description of that theme/challenge (Column 2) and the subsequent changes made or steps taken to address that issue (Column 3)

In the second cycle, we recruited five patients; the mean age was 57 years old, all were female, and REALM-SF literacy scores ranged from 0 to 8 with 60% (*n* = 3) of patients scoring as low literacy. We identified four new themes/challenges: unclear how to select answer, audio button unused or misinterpreted, confusion with scrolling, and incomplete popup window missed. A description of these new themes/challenges, in addition to the corresponding changes that were made to the multimedia PROMIS-UE, are summarized in Table 3.

**Table 3:** Themes/challenges from cycle 2 of qualitative evaluation and corresponding changes

Theme/Challenge	Description	Changes to Multimedia PROMIS-UE
Unclear how to select answer	Uncertainty with how to select answer; some clicked the response audio thinking this was the way to select the response	Added one line of instruction: “Press (audio button) to hear the words”; put audio button in shaded box to differentiate from the rest of the questionnaire; added radio buttons that are more traditionally used for selecting an answer
Audio button unused or misinterpreted	Audio button unused even for low literacy cohort; when used, seemed to be accidental (e.g., thought it was the way to submit an answer or thought it was for volume)	
Confusion with scrolling	Didn’t understand the need to scroll to proceed to the next question	Made every page its own question so that no scrolling is needed; added an arrow button to proceed to the next question
Incomplete popup window missed	Did not notice popup window notifying them of unanswered question	Made the popup window appear front and center on the screen so that it cannot be missed

Legend: This table includes the four additional themes/challenges (Column 1) that we identified in our second cycle of qualitative evaluation, as well as a more detailed description of that theme/challenge (Column 2) and the subsequent changes made or steps taken to address that issue (Column 3)

There were no issues identified surrounding the medium skin tone and gender-neutral person used in the animations, so we elected to proceed without the avatars due to the confusion that they generated. For the audio icon, all patients liked the alternatives better than the current version which was a speaker (Fig. 3), so we switched it to an icon of an ear. For the submit button, we proceeded with an arrow and “FINISH.”

**Fig. 4:**
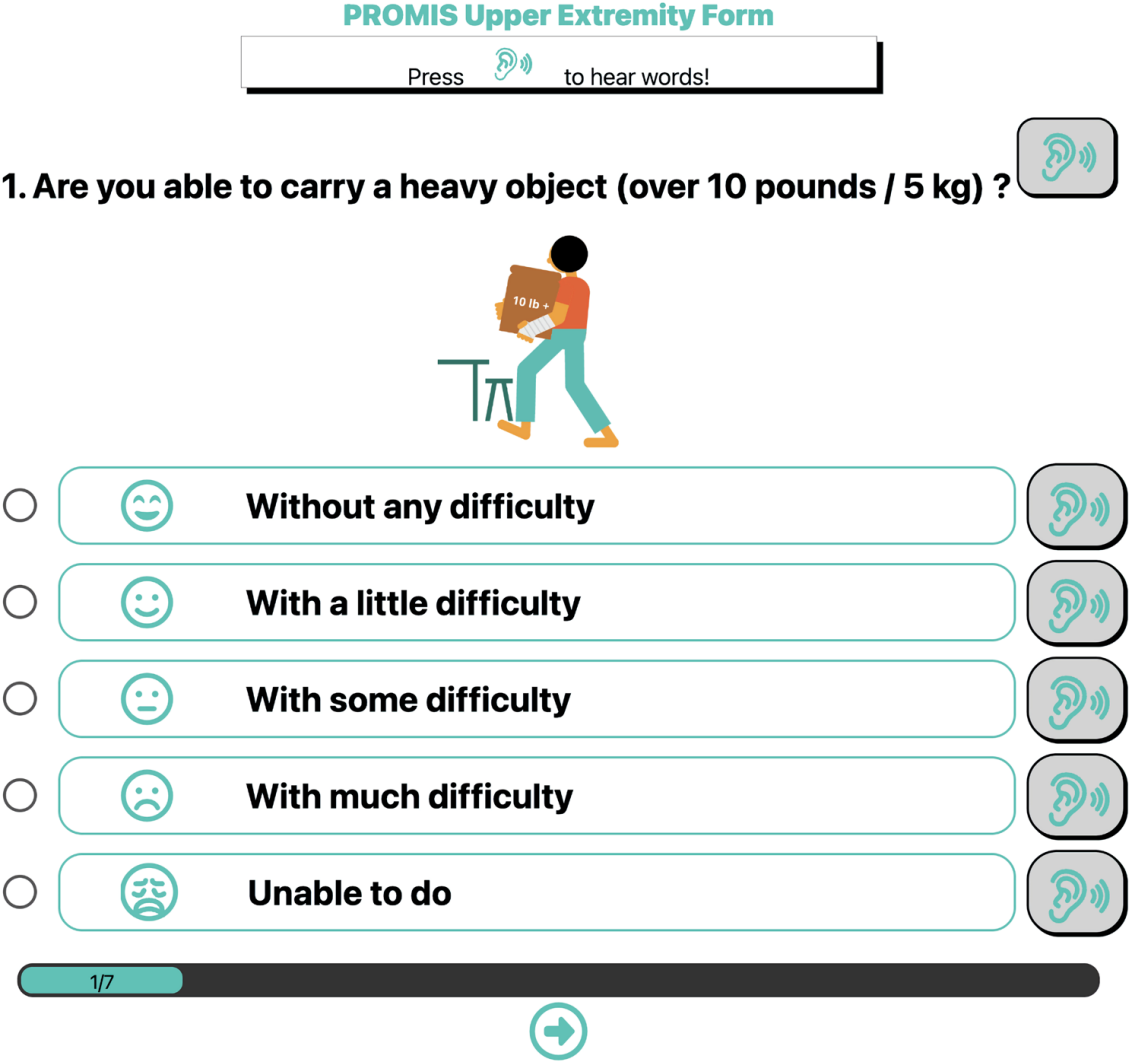
**Screen capture of the final multimedia PROMIS-UE. **This is a screen capture of the first question of the multimedia PROMIS-UE, illustrating the final interface and some of the updated features as summarized in Table 2

The final version of the multimedia PROMIS-UE can be accessed online [36] or seen in the representative screen captures in Fig. 4.

## Discussion

Because almost all PROMs are text-based, precluding self-administration by patients with low literacy, the MAP was designed to adapt text PROMs to multimedia versions that can be self-administered by patients of all literacy levels [16]. The MAP includes four stages: forward adaptation, back adaptation, qualitative evaluation, and psychometric validation. We used the MAP in a hand and upper extremity population to generate a multimedia version of the PROMIS-UE. We previously reported the forward adaptation of PROMIS-UE to a multimedia PROMIS-UE prototype [17]. In this paper, we refined the multimedia PROMIS-UE prototype via back adaptation and qualitative evaluation, which resulted in a conceptually-equivalent, user-tested multimedia PROMIS-UE. Because this was the first attempt to execute the MAP, this study demonstrates the feasibility of adapting text PROMs to rigorously tested multimedia versions without compromising question intent.

Although previous work has developed PROMs that have audio read-aloud features (e.g., the “Talking Touchscreen” [37]), we expand the concept of multimedia PROMs by adding visuals. While other types of instruments (e.g., patient education tools, scales) have been adapted to include audiovisuals [38, 39], this has not been attempted for PROMs. Leveraging multiple mediums of communication can improve comprehension and recall, minimize cognitive burden, and broaden accessibility to low literacy and other populations [38].

Our phased approach, including iterative testing and development, contributes to the robustness of this final multimedia PROMIS-UE. Because the goal was to create an adaptation of the PROMIS-UE rather than to create a new PROM, back adaptation allowed us to ensure that we preserved the content of the PROMIS-UE, much like what is needed when translating PROMs to different languages. Additionally, our iterative qualitative evaluation allowed us to address patient-identified usability, functionality, and content-related issues. Because the central goal of the multimedia PROMIS-UE is to capture PROs from patients with low literacy who have disproportionally lower education attainment and technology literacy, a well-designed multimedia PROMIS-UE that has been thoroughly tested in this target user population will be critical to successful implementation, long-term adoption, and scalability. Poorly designed user interfaces compromise the likelihood of adoption and introduce the risk of human error and operating inefficiencies even for high-functioning, well-trained users [34].

This conceptually-equivalent, user-tested multimedia PROMIS-UE is ready to undergo the final stage of the MAP, psychometric testing, and validation. This will evaluate the multimedia PROMIS-UE’s construct validity, internal consistency, content validity, and responsiveness. Future validation efforts can also evaluate PROMIS-UE and multimedia PROMIS-UE’s differential item functioning across literacy levels. We believe that the robust development of the refined multimedia PROMIS-UE increases the likelihood that the multimedia PROMIS-UE will demonstrate similar or better psychometric properties to the PROMIS-UE, which is essential before implementation can proceed in clinical practice, quality improvement efforts, or research. Ultimately, we plan to expand this work by adapting additional text PROMs to mPROMs in broader patient populations. While the animations/GIFs specific to each question would need to be re-developed and retested via the MAP, some design elements such as the illustrated response scale or the mPROM interface could be reused.

The primary limitation to our work presented in this manuscript, inherent to any iterative design methodology, is that it is always possible to conduct more cycles of testing that may reveal additional issues. In our two cycles of qualitative testing, we identified 12 usability and content-related issues in the first cycle and 4 in the second cycle, demonstrating a significant improvement between the two cycles. In these two cycles, we gathered the information needed to guide the design decision-making relevant to our research goals. We did not expect that additional cycles of testing would reveal major issues that we would need to or elect to address. Additionally, the PROMIS-UE is written above the sixth-grade reading level, so the impact of adapting other questionnaires that are already written at or below the sixth-grade reading level may be diminished. Finally, the testing in this study was performed in a research setting rather than in a real-life clinical setting, so it does not evaluate implementation considerations. We plan to conduct formal implementation testing after the multimedia PROMIS-UE has undergone psychometric validation.

## Conclusions

We executed and demonstrated the feasibility of the back adaptation and qualitative evaluation stages of the MAP by refining the multimedia PROMIS-UE prototype to a conceptually-equivalent, user-tested multimedia PROMIS-UE. This multimedia PROMIS-UE is ready to undergo psychometric testing and validation. A psychometrically validated multimedia PROMIS-UE will expand clinicians’ and investigators’ abilities to capture PROs in hand and upper extremity patients of all literacy levels. Ultimately, a multimedia approach may be applied to other PROMs and broader patient populations, improving the accessibility of PROMs and shifting us to a more equitable paradigm in PRO measurement.

## Data Availability

The datasets generated and/or analysed during the current study cannot be shared publicly to protect patient privacy and comply with ethical or legal requirements. Audio recordings cannot be anonymized due to a person’s voice constituting a unique biometric identifier, and the content of the recordings also contains information that may compromise patient anonymity.

## Abbreviations

CNHC: Curtis National Hand Center
GIF: Graphics Interchange Format
HCD: Human–Centered Design
MAP: Multimedia Adaptation Protocol
mPROM: Multimedia Patient–Reported Outcome Measure
Multimedia PROMIS: UE–Multimedia Patient–Reported Outcomes Measurement Information System Upper Extremity
PROM: Patient–Reported Outcome Measure
PROMIS: UE–Patient–Reported Outcomes Measurement Information System Upper Extremity Short Form
PRO: Patient–Reported Outcome
REALM: SF–Rapid Estimate of Adult Literacy in Medicine–Short Form
UE: Upper Extremity
US: United States

## References

[1] Arozullah AM, Yarnold PR, Bennett CL, Soltysik RC, Wolf MS, Ferreira RM et al (2007) Development and validation of a short-form, rapid estimate of adult literacy in medicine. Med Care 45(11):1026–1033. 10.1097/MLR.0b013e3180616c1b 18049342 10.1097/MLR.0b013e3180616c1b

[2] Bass PF 3rd, Wilson JF, Griffith CH (2003) A shortened instrument for literacy screening. J Gen Intern Med 18(12):1036–1038. 10.1111/j.1525-1497.2003.10651.x 10.1111/j.1525-1497.2003.10651.x PMC1494969 14687263

[3] Yost KJ, Webster K, Baker DW, Jacobs EA, Anderson A, Hahn EA (2010) Acceptability of the talking touchscreen for health literacy assessment. J Health Commun 15 Suppl 2(Suppl 280–92. 10.1080/10810730.2010.500713 10.1080/10810730.2010.500713 PMC3269098 20845195

[4] Kirsch IS, Jungeblut A, Jenkins L, Kolstad A (2002) Adult Literacy in America: A First Look at the Findings of the National Adult Literacy Survey. National Center for Education Statistics, Washington

[5] Adult Literacy in the United States (2019) National Center for Education Statistics, Washington. https://nces.ed.gov/pubs2019/2019179/index.asp. Accessed 16 Sep 2020

[6] Weiss BD, Hart G, McGee DL, D’Estelle S (1992) Health status of illiterate adults: relation between literacy and health status among persons with low literacy skills. J Am Board Fam Pract 5(3):257–264 1580173

[7] Rudd RE (2007) Health literacy skills of U.S. adults. Am J Health Behav 31(Suppl 1):S8–18. 10.5555/ajhb.2007.31.supp.S8 17931141 10.5555/ajhb.2007.31.supp.S8

[8] Long C, Beres LK, Wu AW, Giladi AM (2022) Patient-level barriers and facilitators to completion of patient-reported outcomes measures. Qual Life Res 31(6):1711–1718. 10.1007/s11136-021-02999-8 34533759 10.1007/s11136-021-02999-8

[9] Sisodia RC, Rodriguez JA, Sequist TD (2021) Digital disparities: lessons learned from a patient reported outcomes program during the COVID-19 pandemic. J Am Med Inf Assoc 28(10):2265–2268. 10.1093/jamia/ocab138 10.1093/jamia/ocab138 PMC8344913 34244760

[10] Adjei Boakye E, Nair M, Al-Antary N, Wilson C, Kerr K, Zatirka TM et al (2025) Exploratory analysis of electronic patient-reported outcomes collection: comparing online and in-clinic modalities in cancer care. Qual Life Res 34(7):2113–2121. 10.1007/s11136-025-03975-2 40237928 10.1007/s11136-025-03975-2 PMC12182510

[11] Cella D, Hahn EA, Jensen SE, Butt Z, Nowinski C, Rothrock N (2012) Methodological Issues in the Selection, Administration and Use of Patient-Reported Outcomes in Performance Measurement in Health Care Settings. National Quality Forum, Washington

[12] Spilker B (1995) Quality of Life and Pharmacoeconomics in Clinical Trials. Lippincott Williams & Wilkins, Baltimore

[13] Baker DW, Parker RM, Williams MV, Pitkin K, Parikh NS, Coates W et al (1996) The health care experience of patients with low literacy. Arch Fam Med 5(6):329–334. 10.1001/archfami.5.6.329 8640322 10.1001/archfami.5.6.329

[14] Parikh NS, Parker RM, Nurss JR, Baker DW, Williams MV (1996) Shame and health literacy: the unspoken connection. Patient Educ Couns 27(1):33–39. 10.1016/0738-3991(95)00787-3 8788747 10.1016/0738-3991(95)00787-3

[15] Calvert MJ, Cruz Rivera S, Retzer A, Hughes SE, Campbell L, Molony-Oates B et al (2022) Patient reported outcome assessment must be inclusive and equitable. Nat Med 28(6):1120–1124. 10.1038/s41591-022-01781-8 35513530 10.1038/s41591-022-01781-8

[16] Long C, Beres LK, Wu AW, Giladi AM (2021) Developing a protocol for adapting multimedia patient-reported outcomes measures for low literacy patients. PLoS ONE 16(6):e0252684. 10.1371/journal.pone.0252684 34086774 10.1371/journal.pone.0252684 PMC8177453

[17] Azad CL, Beres LK, Wu AW, Fong A, Giladi AM (2024) Developing a multimedia patient-reported outcomes measure for low literacy patients with a human-centered design approach. PLoS ONE 19(6):e0304351. 10.1371/journal.pone.0304351 38838037 10.1371/journal.pone.0304351 PMC11152264

[18] Summary Table for NIH CDE Initiatives (2020) National Library of Medicine, Bethesda. nlm.nih.gov/cde/summary_table_1. Accessed September 18

[19] Item RT, Health Measures (2020) Chicago. https://healthmeasures.net/resource-center/measurement-science/item-response-theory. Accessed September 18

[20] Hays RD, Lipscomb J (2007) Next steps for use of item response theory in the assessment of health outcomes. Qual Life Res 16:195–199. 10.1007/s11136-007-9175-7 17351825 10.1007/s11136-007-9175-7

[21] Fries JF, Krishnan E, Rose M, Lingala B, Bruce B (2011) Improved responsiveness and reduced sample size requirements of PROMIS physical function scales with item response theory. Arthritis Res Ther 13(5):R147. 10.1186/ar3461 21914216 10.1186/ar3461 PMC3308075

[22] PROMIS^®^Instrument Development and Validation Scientific Standards Version 2.0 (2013) HealthMeasures, Chicago. https://www.healthmeasures.net/images/PROMIS/PROMISStandards_Vers2.0_Final.pdf. Accessed April 14, 2026

[23] Shipp MM, Thakkar MY, Sanghavi KK, Means KR Jr, Giladi AM (2021) Disparities Limit the Effect and Benefit of a Web-Based Clinic Intake System. Orthopedics 44(3):e434–e439. 10.3928/01477447-20210415-02 34039210 10.3928/01477447-20210415-02

[24] : County Health Rankings & Roadmaps, City B (2019) MD University of Wisconsin Population Health Institute, Madison. https://www.countyhealthrankings.org/explore-health-rankings/maryland/baltimore-city?year=2019. Accessed 21 Nov 2019

[25] Data USA: Baltimore, MD Data USA, Cambridge (2014) https://datausa.io/profile/geo/baltimore-md/. Accessed 7 Mar 2020

[26] Multimedia PROMIS-UE, Prototype https://mi2apps.medstar.net/mprom/20210806/index.html. Accessed 21 Aug 2021

[27] Macefield R (2009) How To Specify the Participant Group Size for Usability Studies: A Practitioner’s Guide. J Usability Stud 5(1):34–45

[28] Averill JB (2002) Matrix analysis as a complementary analytic strategy in qualitative inquiry. Qual Health Res 12(6):855–866. 10.1177/104973230201200611 12109729 10.1177/104973230201200611

[29] The Field Guide to Human-Centered Design (2021) IDEO.org, San Francisco. https://www.designkit.org/resources/1.html. Accessed 19 Feb 2021

[30] Nielsen J (2000) Why You only need to test with 5 Users. Nielsen Norman Group, Fremont. https://www.nngroup.com/articles/why-you-only-need-to-test-with-5-users/. Accessed 19 Feb 2021

[31] Bazzano AN, Martin J, Hicks E, Faughnan M, Murphy L (2017) Human-centred design in global health: A scoping review of applications and contexts. PLoS ONE 12(11):e0186744. 10.1371/journal.pone.0186744 29091935 10.1371/journal.pone.0186744 PMC5665524

[32] Willis GB (2005) Cognitive Interviewing: A Tool For Improving Questionnaire Design. SAGE, Thousand Oaks

[33] Chierichetti F, Kumar R, Tomkins A (2022) On the number of trials needed to distinguish similar alternatives. Proc Natl Acad Sci U S A 119(31):e2202116119. 10.1073/pnas.2202116119 35901210 10.1073/pnas.2202116119 PMC9351503

[34] Zhang J, Johnson TR, Patel VL, Paige DL, Kubose T (2003) Using usability heuristics to evaluate patient safety of medical devices. J Biomed Inf 36(1–2):23–30. 10.1016/s1532-0464(03)00060-1 10.1016/s1532-0464(03)00060-1 14552844

[35] Nielsen J, Mack RL (1994) Usability Inspection Methods. Wiley, Hoboken

[36] Multimedia PROMIS-UE, Final Version (2024). https://shrey94.github.io/MPROM/Question_1.html. Accessed 1 Jan

[37] HahnEA, Cella D, Dobrez D, Shiomoto G, Marcus E, Taylor SG, et al (2004) The talking touchscreen: a new approach to outcomes assessment in low literacy. Psychosoc 13(2):86-95. Epub 2004/02/12. 10.1002/pon.719. PubMed PMID: 14872527. 10.1002/pon.719 10.1002/pon.719 14872527

[38] GhiassiR, Murphy K, Cummin AR, Partridge MR (2011) Developing a pictorial Epworth Sleepiness Scale. Thorax 66(2):97-100. Epub 2010/10/28. 10.1136/thx.2010.136879. PubMed PMID: 20978025. 10.1136/thx.2010.136879 10.1136/thx.2010.136879 20978025

[39] Lopez-OlivoMA, Ingleshwar A, Volk RJ, Jibaja-Weiss M, Barbo A, Saag K, et al (2018) Development and pilot testing of multimedia patient education tools for patients With knee osteoarthritis, osteoporosis, and rheumatoid arthritis. Arthritis Care Res (Hoboken) 70(2):213-20. Epub 2017/05/04. 10.1002/acr.23271. PubMed PMID: 28464546; PubMed Central PMCID: PMCPMC6442741. 10.1002/acr.23271 10.1002/acr.23271 PMC6442741 28464546

